# Photodissociation of aligned CH_3_I and C_6_H_3_F_2_I molecules probed with time-resolved Coulomb explosion imaging by site-selective extreme ultraviolet ionization

**DOI:** 10.1063/1.4998648

**Published:** 2018-01-25

**Authors:** Kasra Amini, Evgeny Savelyev, Felix Brauße, Nora Berrah, Cédric Bomme, Mark Brouard, Michael Burt, Lauge Christensen, Stefan Düsterer, Benjamin Erk, Hauke Höppner, Thomas Kierspel, Faruk Krecinic, Alexandra Lauer, Jason W. L. Lee, Maria Müller, Erland Müller, Terence Mullins, Harald Redlin, Nora Schirmel, Jan Thøgersen, Simone Techert, Sven Toleikis, Rolf Treusch, Sebastian Trippel, Anatoli Ulmer, Claire Vallance, Joss Wiese, Per Johnsson, Jochen Küpper, Artem Rudenko, Arnaud Rouzée, Henrik Stapelfeldt, Daniel Rolles, Rebecca Boll

**Affiliations:** 1 The Chemistry Research Laboratory, Department of Chemistry, University of Oxford, Oxford OX1 3TA, United Kingdom; 2 Deutsches Elektronen–Synchrotron DESY, 22607 Hamburg, Germany; 3 Max Born Institute for Nonlinear Optics and Short Pulse Spectroscopy, 12489 Berlin, Germany; 4 Department of Physics, University of Connecticut, Storrs, Connecticut 06269, USA; 5 Department of Chemistry, Aarhus University, 8000 Aarhus C, Denmark; 6 Helmholtz Zentrum Dresden Rossendorf, 01328 Dresden, Germany; 7 Center for Free–Electron Laser Science, Deutsches Elektronen–Synchrotron DESY, 22607 Hamburg, Germany; 8 Center for Ultrafast Imaging, Universität Hamburg, 22761 Hamburg, Germany; 9 Institut für Optik und Atomare Physik, Technische Universität Berlin, 10623 Berlin, Germany; 10 Max Planck Institute for Biophysical Chemistry, 33077 Göttingen, Germany; 11 Institute for X-ray Physics, Göttingen University, 33077 Göttingen, Germany; 12 Department of Physics, Lund University, 22100 Lund, Sweden; 13 Department of Physics, Universität Hamburg, 22761 Hamburg, Germany; 14 J. R. Macdonald Laboratory, Department of Physics, Kansas State University, Manhattan, Kansas 66506, USA

## Abstract

We explore time-resolved Coulomb explosion induced by intense, extreme ultraviolet (XUV) femtosecond pulses from a free-electron laser as a method to image photo-induced molecular dynamics in two molecules, iodomethane and 2,6-difluoroiodobenzene. At an excitation wavelength of 267 nm, the dominant reaction pathway in both molecules is neutral dissociation via cleavage of the carbon–iodine bond. This allows investigating the influence of the molecular environment on the absorption of an intense, femtosecond XUV pulse and the subsequent Coulomb explosion process. We find that the XUV probe pulse induces local inner-shell ionization of atomic iodine in dissociating iodomethane, in contrast to non-selective ionization of all photofragments in difluoroiodobenzene. The results reveal evidence of electron transfer from methyl and phenyl moieties to a multiply charged iodine ion. In addition, indications for ultrafast charge rearrangement on the phenyl radical are found, suggesting that time-resolved Coulomb explosion imaging is sensitive to the localization of charge in extended molecules.

## I. INTRODUCTION

If several electrons are rapidly removed from a molecule, it fragments into cations by a process termed Coulomb explosion.^1^ Provided that the break-up occurs faster than vibrational motion, the momenta of the fragments can be used to determine the structure of gas-phase molecules. Coulomb explosion induced by intense femtosecond (fs) laser pulses in the visible or the near-infrared (NIR) region^2^ can be used as a time-resolved structural probe of molecular dynamics. A molecular reaction, such as photodissociation, is initiated with a fs pump pulse, and the evolving structure is measured as a function of time using a delayed, intense Coulomb explosion pulse. Such time-resolved Coulomb explosion imaging (CEI) has been used to study photoisomerization,^3,4^ photodissociation,^5–7^ and torsional motion in an axially chiral molecule.^8,9^ An alternative method for inducing Coulomb explosion employs irradiation with extreme ultraviolet (XUV) or X-ray femtosecond pulses, notably from intense free-electron laser (FEL) sources.^10–14^ Several recent experiments have demonstrated the feasibility of studying molecular dynamics such as fragmentation,^15^ isomerization,^16,17^ charge transfer,^18,19^ and interatomic Coulombic decay^20^ in real time.

XUV or X-ray induced Coulomb explosion differs from its strong-field induced equivalent in several respects: While strong-field ionization removes electrons from the molecular valence shell, which is typically highly delocalized, the XUV or X-ray photon energy can be tuned to an inner-shell absorption edge, thus making the photoabsorption site- and element specific. Furthermore, the kinetic energies (KE) and angular correlations of the ionic fragments resulting from strong-field induced Coulomb explosion typically strongly depend on the pulse duration,^6,21,22^ while this dependence can be less pronounced in the case of inner-shell ionization, where the time-scale of the Auger decay is often the most relevant parameter, especially for Coulomb explosion induced by single-photon absorption.

**FIG. 1.**
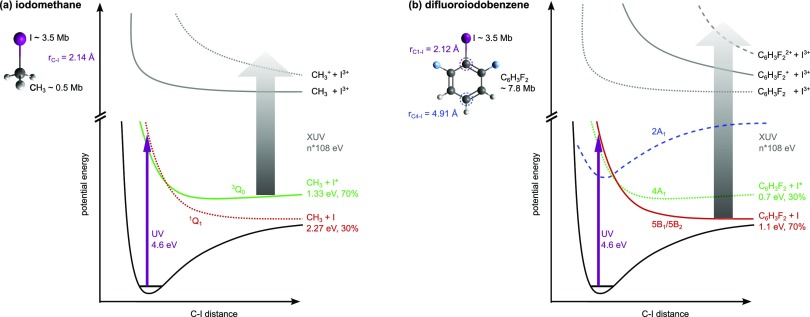
Schematic one-dimensional potential energy curves (PECs) of (a) iodomethane and (b) difluoroiodobenzene. The PECs corresponding to the dominant channels in our experiment are shown as solid lines. The dissociative states, ^3^Q_0_ and ^1^Q_1_ in CH_3_I and 4A_1_ and 5B_1_/5B_2_ in DFIB, are shown as red and green lines and are very similar for both molecules (the nomenclature follows Ref. 26). The total kinetic energy release of the resulting products and their asymptotic relative populations after absorption of one 267 nm photon (purple arrow) are shown.^23,26,27^ In DFIB, an additional predissociative state (dashed blue) can also be populated. The XUV probe pulse (grey arrow) promotes the system to one of the many multiply charged potential energy curves through single or multi-photon absorption. For simplicity, only exemplary curves leading to triply charged iodine ions in the final state are shown here. The sum of the atomic cross-sections for single-photon ionization at a photon energy of 108 eV is indicated next to the sketch of the molecules.^28^

Here, we focus on the role of site-selective ionization in time-resolved Coulomb explosion imaging experiments. To this end, we investigate the ultraviolet (UV)-induced photoexcitation and subsequent XUV ionization and fragmentation of isolated iodomethane (CH_3_I) and 2,6-difluoroiodobenzene (C_6_H_3_F_2_I, DFIB) molecules, see Fig. 1. The photochemistry of iodomethane in the A-band (210–350 nm) has been the subject of previous experimental and theoretical studies, see, for example, Refs. 23–25 and references therein. In this energy range, the photoexcitation (purple arrow in Fig. 1) triggers almost exclusively a resonant one-photon dissociation into two neutrals, by promoting an electron to the σ* orbital along the C–I bond. This results either in ground state iodine, CH_3_ + I, with a yield of ∼30%, or in spin-orbit excited iodine, CH_3_ + I^*^, with a yield of ∼70%,^23^ as illustrated in Fig. 1(a). The UV-photochemistry of fluorinated aryl iodides is less well studied but can be regarded as largely similar to the case of iodobenzene.^26^ Two channels analogous to the case of CH_3_I are accessible through single-photon UV excitation but lead to opposite yields in the two spin-orbit components (∼70% I, ∼30% I^*^).^27^ In addition, bound states involving electron density on the phenyl ring are overlapped with the A-band (240–320 nm) and can thus form a predissociative state by mixing with the C–I dissociative state, see the dashed blue line in Fig. 1(b). Creation of ground-state iodine atoms via the predissociative channel is strongly suppressed as compared to the direct dissociation.^26^

The similarity of the UV-pump step for both molecules gives us the opportunity to study the role of site-selective ionization and the influence of the molecular environment, i.e., methyl versus phenyl moiety, on the XUV-probe step (grey arrow in Fig. 1). At a certain time delay after the UV excitation, the dissociating molecule is ionized by the free-electron laser probe pulse, leading to a highly excited molecular ion that fragments through Coulomb explosion. This is illustrated in Fig. 1, showing one-dimensional cuts through the potential energy hypersurfaces of the multiply charged molecular ion which are formed following (multiple) ionization of the excited molecules by the FEL pulse. Note that absorption of more than one XUV photon in the same molecule is possible in our experiment, due to the high peak intensity of the XUV pulse in the focus. The potential energy curves (PECs) involving three or more charges on the iodine, as illustrated in Fig. 1, are created by absorption of two or more XUV photons. While the XUV-ionization can be regarded as site-selective in CH_3_I, as ∼90% of the absorbed photons are absorbed at the iodine atom at a photon energy of 108 eV, in DFIB, ∼70% of the absorbed photons are absorbed at the difluorobenzene (DFB) radical.^28^

## II. EXPERIMENT

The experiment was carried out in the CAMP endstation^29,30^ at the Free-Electron Laser in Hamburg (FLASH).^31^ The experimental setup as well as the data treatment has been described in detail in Refs. 32 and 33. In brief, CH_3_I or C_6_H_3_F_2_I molecules were mixed with neon (20 bars) at room temperature and supersonically expanded through a pulsed Even-Lavie valve (opening time 12.5 *μ*s) and then passed through two skimmers. An electrostatic deflector positioned between the skimmers selected the lowest-lying rotational quantum states and also partially separated the molecules from the neon, as the atomic carrier gas is unaffected by the deflector. This increases the maximum degree of molecular alignment that can be achieved^34,35^ and, moreover, reduces significantly the amount of background ions from the neon carrier gas and thus prevents detector saturation. In the interaction region, the molecular beam was intersected by the free-electron laser (FEL) and two additional laser beams, which propagated collinearly to the FEL. A near-infrared (NIR) pulse from an Nd:YAG laser [1064 nm, 12 ns (FWHM), 1.2 J, 50 *μ*m focus (FWHM)] was used to adiabatically align the molecules^36^ such that their most polarizable axes, the C–I axes, were aligned along the laser polarization direction, parallel to the detector plane. At the peak of the alignment pulse, where the degree of alignment is highest,^36^ the molecules were first photoexcited by an ultraviolet laser pulse [267 nm, 150 fs (FWHM), 35 *μ*J, 50 *μ*m focus (FWHM)] and then probed by an intense extreme-ultraviolet FEL pulse [108 eV, 120 fs (FWHM), 37 *μ*J on average, 20 *μ*m focus (FWHM)] after a tunable time delay. The polarizations of the UV and the FEL pulse were parallel to each other, in the detection plane. The repetition rate of the experiment was 10 Hz. The delay between the UV pump pulse and the XUV probe pulse was set using a motorized delay stage in the UV arm. The data were acquired by recording 1000 shots per delay step of 83 fs in a range of ±1 ps, i.e., 24 000 shots for all CH_3_I data. For DFIB, the delay step size was 66 fs for I^2+^, 20 fs for I^3+^, and 53 fs for I^4+^, and the total number of shots contained in the delay scans of the different ion species was 30 000, 105 000, and 15 000, respectively. In the data analysis, these shots are resorted and rebinned according to the information from the beam arrival time monitor of the electron bunch.^32^

Ions and electrons resulting from the photoionization were recorded simultaneously using a double-sided velocity-map imaging (VMI) spectrometer^29,37^ by multichannel plates coupled to phosphor screens. The corresponding two-dimensional ion momentum distributions were recorded using a commercial one-Megapixel CCD camera (Allied Vision Pike F-145B) or the 72 × 72 pixel time-stamping Pixel Imaging Mass Spectrometry camera (PImMS).^38,39^ The cameras were mounted outside of vacuum and could be interchanged easily such that pump-probe scans were recorded for both molecules with both cameras. The CCD camera provides a much higher spatial resolution, but it does not have the timing resolution to distinguish between the different ionic species that arrive at the detector with flight-time differences of several hundred nanoseconds after a total flight time of a few microseconds. Therefore, the high voltage on the MCP detector was gated using a fast high-voltage switch such that only a specific ion species was detected at a time, and the pump-probe scans for different ion species were recorded consecutively. The PImMS camera, on the other hand, can record and time-stamp up to four ion hits per pixel with a 12.5 ns timing precision, which is sufficient to distinguish the different iodine charge states and most other ionic species in this experiment (the corresponding time-of-flight spectra and further details are given in Ref. 33). With the PImMS camera, the yields and 2D momentum distributions of all ionic species can therefore be recorded within the *same* pump-probe scan, albeit with a lower spatial resolution than with the CCD camera.

In the subsequent data analysis, the 2D momentum distributions of each ionic species recorded with the CCD or the PImMS camera were angularly integrated, and the radii were then converted into kinetic energies (KE) based on ion trajectory simulations carried out using the Simion 8.0 software package, from which an empirical formula was constructed that connects the hit position on the detector with the fragment's kinetic energy. Further discussion of the observed ion kinetic energies in the Coulomb explosion of DFIB is also given in Ref. 33. Here, we concentrate on discussing the yields and kinetic energies of the multiply charged iodine fragments from CH_3_I and DFIB and, in particular, their dependence on the delay between the UV and XUV pulses. The iodine ion KE distributions were converted to a total kinetic energy release (TKER) based on the assumption that the cofragment is momentum-matched with the recorded iodine ion. This assumption is exact for a two-body fragmentation, as is induced by the UV pulse. It is expected to also apply to the majority of XUV ionization events that occur in already dissociated molecules.

Note that by using strong, adiabatic alignment of the C–I axes parallel to the detector plane, the component of the ion momentum along the spectrometer axis is effectively confined to zero such that the recorded radial distribution of iodine ions on the detector corresponds to the momentum distribution to a very good approximation, thus making image inversion algorithms that are normally used in VMI spectroscopy unnecessary in this case.^40^ The degree of alignment determined from the I^3+^ ion images was $〈\;{\text{cos}}^{2}\Theta_{2D}〉=0.92$ for CH_3_I and 0.94 for DFIB, corresponding to standard deviations of 17° and 15° with respect to the XUV polarization direction, respectively.

Adiabatic alignment is an alternative to the retrieval of the molecular structure by coincident ion momentum spectroscopy employing delay-line anodes. Our approach allows for experimental conditions with high ion count rates per shot when an MCP/phosphor screen detector is used. Molecular alignment parallel to the detection plane is particularly powerful in combination with a time-stamping camera such as PImMS^38,39^ or TimepixCam^41,42^ or an in-vacuum pixel detector,^43^ facilitating the recording of all ionic species simultaneously. These images provide the opportunity to study angular correlations between different ionic fragments, which, with the help of laser alignment, can be interpreted in a straightforward way, allowing detailed conclusions to be drawn about structure and fragmentation dynamics.^9,44–49^

## III. RESULTS

Details of the static Coulomb explosion imaging of laser-aligned DFIB molecules as well as more technical aspects of the laser/FEL pump-probe data analysis have been discussed before.^32,33^ In the present manuscript, we focus on the molecular dynamics due to the UV-dissociation and the subsequent XUV-induced Coulomb explosion and on the comparison between DFIB and CH_3_I molecules.

**FIG. 2.**
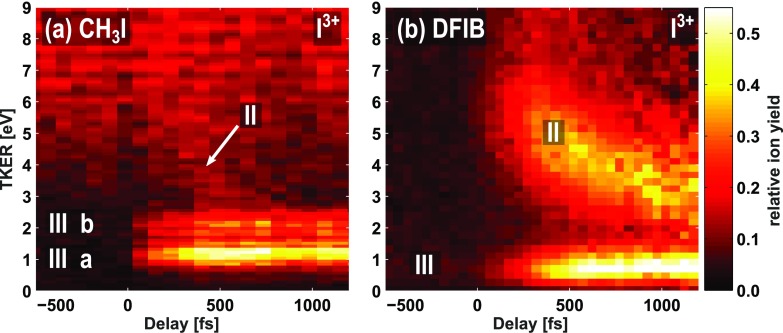
Low-kinetic-energy region of the total kinetic energy release of triply charged iodine ions as a function of the delay between the UV-pump and XUV-probe pulses for (a) iodomethane and (b) difluoroiodobenzene molecules, recorded using the CCD camera. Negative delays correspond to the XUV pulse arriving first and positive delays to the UV pulse arriving first, in accordance with our earlier, related publications.^18,19^ Different fragmentation channels, II and III, are indicated and are discussed in the main text.

**FIG. 3.**
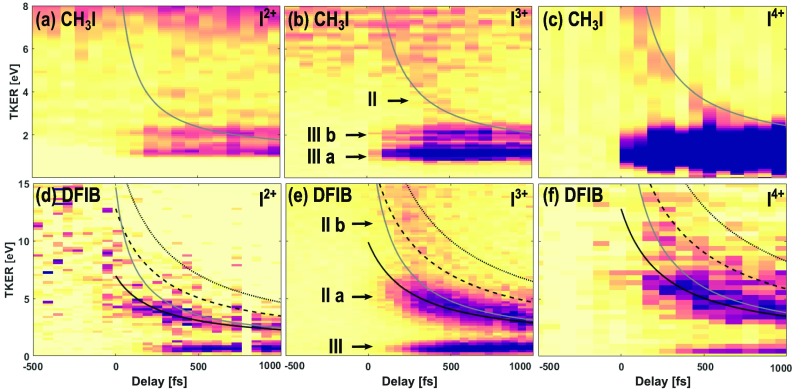
Delay-dependent total kinetic energy release of different iodine charge states arising after UV-excitation and subsequent XUV ionization of aligned CH_3_I (top) and DFIB (bottom) molecules, recorded using the CCD camera. The results of Coulomb explosion simulations are superimposed as lines. Solid lines correspond to an I^*n*+^ ion dissociated with a singly charged molecular rest and dashed lines to a doubly and dotted lines to a triply charged partner. For CH_3_I, a C–I distance of 2.14 Å has been used for the calculation, and for DFIB, r_C1–I_ = 2.12 Å (grey) and r_C4–I_ = 4.91 Å (black) are displayed. The DFIB data were normalized to the sum of the FEL pulse energy in each delay bin, and a jitter-correction was applied.^32^ For the CH_3_I dataset, the beam arrival time monitor was not operational, and single-shot information was not collected. Therefore, the jitter could not be corrected and the ion yield was normalized to the number of acquisitions. Given the FEL and laser pulse durations in this experiment, the influence of the arrival time jitter between laser and FEL pulses, which is typically less than 200 fs (FWHM), should not significantly broaden the effective pump-probe instrument response function. In (d) and (f), the UV late spectrum (delays ≤ −270 fs) has been subtracted from all delay bins to provide better visibility. In (c), no centroiding could be applied and no normalization was carried out.

As described in the introduction, the dominant process upon UV-excitation of both molecules is neutral C–I bond cleavage. In the following, we describe how the XUV-probe signal resulting from the dissociated molecules depends on the molecular environment. At the intensity used in this experiment, ionization by the UV laser pulse and the Nd:YAG laser pulse alone was minimal and, in particular, neither laser pulse produced any multiply charged ions. Figures 2(a) and 2(b) show the low-energy region of the delay-dependent total kinetic energy releases determined for the two molecules from the triply charged iodine ions. Two dynamic features are visible: a strong feature (III), which has a TKER independent of the delay, and a second feature (II), which is strong in DFIB and weak in CH_3_I and corresponds to a TKER that varies as a function of the delay. The numbering of the fragmentation channels follows the nomenclature of our earlier publications.^18,19^ Similar features also appear for I^2+^ and I^4+^, as shown in Fig. 3. Outside the axis range chosen here, an additional broad feature at higher TKER values is present, corresponding to Coulomb explosion of bound molecules by the XUV pulse alone, as discussed in more detail in Ref. 33.

### A. Local XUV ionization at iodine (channel III)

**FIG. 4.**
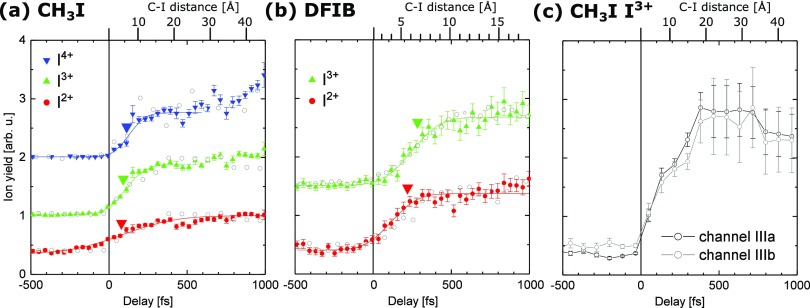
Delay-dependent ion yield in channel III for different iodine charge states from (a) iodomethane and (b) difluoroiodobenzene molecules. The data points are obtained by integrating all recorded ions of each species within a TKER range of 0–2.5 eV for CH_3_I and for a TKER range of 0–1.5 eV for DFIB, recorded using the CCD camera (gray circles) or the PImMS camera (colored symbols). To extract the center of the step functions, a Gaussian cumulative distribution function was fitted to the PImMS data, as shown by the solid lines. For better visibility, the yields for the different charge states are offset on the vertical axis. Negative delays correspond to the XUV pulse arriving first and positive delays to the UV pulse arriving first. The large inverted triangles indicate the calculated critical distances for internuclear charge transfer resulting from the over-the-barrier model (see the text). (c) Delay-dependent ion yield of the two components III a (integrated over the TKER range of 0–1.5 eV) and III b (1.5–2.5 eV) in the I^3+^ channel in CH_3_I, recorded using the CCD camera.

Channel III can be assigned to neutral C–I bond cleavage induced by absorption of one UV photon, followed by XUV-ionization of the isolated iodine atom. The cofragment does not interact with the XUV pulse and remains neutral, and therefore, the resulting TKER in channel III is determined solely by the translational energy gained during the UV-dissociation when the molecule has dissociated into two independent fragments. However, closer inspection of the delay-dependencies in the CH_3_I and C_6_H_3_F_2_I data (Figs. 2 and 3) reveals that these channels are not centered around zero pump-probe delay but are instead shifted towards positive delays. To show this more clearly, Fig. 4 displays the delay-dependent ion yield of channel III for different iodine charge states. The shift in the onset of this channel for both molecules is attributed to the existence of ultrafast intramolecular electron transfer from the methyl or difluorophenyl radical to the multiply charged iodine ion, which cannot happen at large internuclear separations. This process was discussed in detail in Refs. 18 and 19 for iodomethane. If the two moieties are in close proximity to each other, which is the case at small delays between the UV and the XUV pulses, the methyl or difluorophenyl radical will not remain neutral in the vicinity of a multiply charged iodine ion but become singly charged via electron transfer. The corresponding fragment pair will gain additional Coulomb energy and thus will not appear in channel III, but rather at higher TKERs and with an iodine charge state reduced by one.

It was shown in Refs. 18 and 19 that for iodine 3d ionization of iodomethane, the critical internuclear distance up to which charge rearrangement is observed can be well reproduced by a classical over-the-barrier model,^50–52^ which describes the electron transfer as a result of the suppression of the potential barrier between the multiply charged iodine ion and the neutral radical in close proximity. The values for the critical internuclear distance resulting from this classical over-the-barrier model applied to the present case are indicated by the large inverted triangles in Fig. 4.

Before discussing these results further, we would like to note that Fig. 4 shows the delay-dependent ion yields obtained from multiple pump-probe scans using the CCD (gray circles) and the PImMS camera (colored symbols). In the former case, the datasets were measured consecutively over the course of several days of beamtime. Long-term timing drifts, which could not be measured and corrected accurately enough, made it impossible to determine one absolute time zero for all delay scans. Therefore, the time zero was determined for each delay scan individually by matching the simulated Coulomb curves for channel II (see Sec. III B) to the experimental data. The accuracy of this method is of the same order as the temporal resolution of the experiment, which was estimated to be 200 fs (FHWH)^32^ and was limited by the FEL and laser pulse durations.

In contrast, for the datasets recorded using the PImMS camera, the delay-dependence of the different iodine charge states with respect to each other is well defined since all ions were recorded within the same pump-probe run and only the common time zero had to be determined, which was done by fitting channel II in the I^3+^ ion yield. Therefore, the uncertainty of the absolute time-zero determination does not affect the relative differences observed between different charge states. Furthermore, it is worth pointing out that although time zero has been determined *independently* for the data recorded with the PImMS and the CCD camera, the datasets are in good agreement, suggesting that the method for determining time zero is rather robust.

Unfortunately, in the case of DFIB, the 12.5-ns time resolution of the PImMS camera was not sufficient to distinguish the I^4+^ fragment from the nearby CF^+^, which has a mass-to-charge ratio that differs by less than one unit and therefore has a very similar time of flight. Furthermore, the lower spatial resolution of the PImMS camera resulted in a lower kinetic energy resolution such that channels II and III could not be well separated at large delays, most notably in the case of I^4+^ from CH_3_I.

In order to analyze the relative shift in the onset of channel III for each of the iodine charge states, a Gaussian cumulative distribution function (CDF) was fitted to the PImMS data in Fig. 4. For CH_3_I, the CDF fits yield center positions of 87 ± 20 fs, 104 ± 7 fs, and 122 ± 11 fs for I^2+^, I^3+^, and I^4+^, respectively. For DFIB, the center positions are 111 ± 14 fs and 242 ± 13 fs for I^2+^ and I^3+^, respectively. The indicated errors are the statistical errors of the fits. We can thus conclude that the charge transfer process, which was previously observed to occur after iodine 3d ionization of iodomethane, also occurs after iodine 4d ionization of both iodomethane and difluoroiodobenzene, demonstrating that the electron transfer from a neutral molecular fragment to a multiply charged atomic ion as introduced in Refs. 18 and 19 is not particular to iodomethane but also occurs from a phenyl moiety. Furthermore, within the experimental uncertainties and temporal resolution, the classical over-the-barrier-model is consistent with the data. In particular, the onset of channel III appears to be shifted to later delays in DFIB as compared to iodomethane. This can, to a large extent, be explained by pure kinematics when taking into account that the UV-excitation of DFIB preferentially results in a two-body dissociation into an iodine atom (mass 127) and a difluorobenzene radical (DFB, mass 113), i.e., two almost equally heavy partners. By momentum conservation, this leads to a slower dissociation velocity as compared to iodomethane, where the CH_3_ radical (mass 15) gains a much higher kinetic energy. Therefore, the internuclear distance in DFIB increases much slower than in CH_3_I, and the critical distance, at which electron transfer between the two fragments can no longer occur, is reached at larger delays. The top axes in Fig. 4, which indicate the C–I internuclear distance for a given pump-probe delay and were used to position the inverted triangles in this plot, were calculated assuming constant velocities of the neutral fragments created by the UV pulse. These velocities were calculated from the corresponding asymptotic TKER values given in the literature.^23,26^ The assumption of constant velocities is an approximation that overestimates how quickly the internuclear distances increase since the fragments do not reach their asymptotic energy value instantaneously. A more precise model, which shall not be developed here, would thus require quantitative knowledge of the dissociative potential curves in the neutral molecule.

We would also like to point out that without coincident electron spectroscopy and/or detailed quantum chemistry calculations, we cannot draw conclusions about the absolute charge transfer probabilities or any details about the underlying electronic processes that lead to the charge transfer in the two molecules. These processes are expected to differ significantly between the methyl and phenyl radicals, as DFIB has many more electrons, which are partly delocalized over the ring. The influence of the molecular environment on the charge rearrangement in iodomethane and iodobenzene molecules has been recently investigated for ultraintense hard X-rays, both experimentally and theoretically.^53^

Interestingly, in our two earlier X-ray experiments on iodomethane, channel III appeared only for charge states I^4+^ and higher.^18,19^ In the XUV regime however, it can also be observed for I^2+^ and I^3+^ ions but not for I^+^ (not shown). This can be understood when considering the fact that in the X-ray experiments, the iodine M-shell was accessible for ionization, whereas at 108 eV, iodine 4d is the deepest energy level that can be ionized by a single photon.^54^ In the former case, ionization of an isolated iodine atom results predominantly in I^4+^ and I^5+^, as can be concluded by comparison to the M-shell ionization of Xe atoms,^55^ which are isoelectronic to I^–^ and thus have a similar cross-section and Auger relaxation pathways. I^2+^ and I^3+^ are produced only after ionization of the intact molecule and thus do not exhibit low-energy channel III that stems from dissociated molecules. At 108 eV, in contrast, ionization of an isolated iodine atom results predominantly in I^2+^ and I^3+^ (again, we refer to the case of Xe for comparison^55^) while I^+^ is only produced from intact molecules and is thus the only charge state where low-energy channel III does not occur. We note that iodine atoms recorded with four or more charges must be created through absorption of at least two XUV photons, thus confirming that multiphoton absorption plays a significant role in this experiment.

Finally, we would like to investigate the delay-dependence of the two components of channel III, labeled III a and III b in Fig. 2(a), which correspond to the two different spin-orbit components that contribute to the UV excitation, as illustrated in the PECs in Fig. 1. Initially, excitation at 267 nm occurs to the ^3^Q_0_ state, but a fraction of the dissociating wavepacket is transferred to the ^1^Q_1_ potential energy surface via nonadiabatic coupling, resulting in a relative population of roughly 70% in the CH_3_ + I^*^ channel and 30% in the CH_3_ + I channel.^23^ According to Ref. 23, the total kinetic energy release expected for channels III a and III b is 1.33 and 2.27 eV, respectively. The relative ion yield as well as the TKER values of both channels in the present dataset is in accordance with these literature values within the uncertainty of our experimental energy calibration, which is of the order of ∼20%.

As shown in the delay-dependent ion yields in Fig. 4(c), channels III a and III b exhibit the same delay-dependence within the uncertainties of the present measurement, which is to be expected, as the non-adiabatic curve crossing between the ^3^Q_0_ and the ^1^Q_1_ states takes place within <50 fs, i.e., at a very short internuclear distance.^24^ At such early delays, channel III is not yet observed since ultrafast charge rearrangement necessarily results in a charged cofragment at such small internuclear distances. Note that the two sub-components were not resolved in our earlier UV-pump, soft X-ray-probe experiment on CH_3_I,^19^ utilizing a time-of-flight mass spectrometer with a small aperture and without a position-sensitive detector.

For DFIB, channel III comprises two analogous components, with a TKER of 0.7 eV for the ejection of I^*^ and 1.1 eV for the ground state and an I^*^ to I ratio of 30/70 (opposite to the case of CH_3_I).^26^ These components are, however, not resolved in the present data, probably because the DFIB data have been recorded at a factor of four higher spectrometer voltages as compared to CH_3_I in order to allow for simultaneous 4*π* electron detection on the opposite side of the VMI. The mean value of 0.7 eV is in accordance with the published TKER values^26^ within the limited energy resolution of this dataset.

### B. Non-selective XUV ionization of both photofragments (channel II)

We now turn to the feature exhibiting a rapidly decreasing kinetic energy as a function of the pump-probe delay, labeled channel II in Figs. 2 and 3. The decreasing kinetic energy indicates that this channel is the result of a Coulomb repulsion between two *charged* fragments, whose distance increases as the pump-probe delay increases. Given that the pulse intensity was tuned such that almost no ionization was induced by the UV pulse alone and noting that channel II is also present at long pump-probe delays when charge transfer between the two fragments is no longer possible, the origin of this channel must be the absorption of *two* XUV photons, one by each of the fragments.

The observation that channel II is much stronger for DFIB than for CH_3_I for all iodine charge states, relative to the yield in channel III, can be understood as follows: considering the sum of the atomic photoabsorption cross-sections for each of the fragments at a photon energy of 108 eV, ∼90% of the absorbed photons are absorbed by the iodine atom and only ∼10% by the CH_3_ fragment (for the case of iodomethane), whereas in DFIB, ∼70% of the absorbed photons are absorbed by the difluorobenzene radical and only ∼30% by the iodine.^28^ Following the neutral UV-dissociation, it is thus significantly more probable that an XUV photon is absorbed by the DFB radical than by the isolated methyl group. Therefore, channel II is much stronger in DFIB than in iodomethane. We note that channel II, similar to channel III discussed above, also seems to start appearing at slightly positive delays, especially for the higher iodine charge states in DFIB. The most likely reason is again an ultrafast charge transfer process, by which an electron from the singly charged methyl fragment is transferred to the highly charged iodine ion when the latter is in very close proximity, thereby increasing the total Coulomb energy of this fragment pair.

In order to further investigate the origin of channel II and to assign it to a specific Coulomb explosion channel, numerical 2-step Coulomb explosion simulations have been carried out. The fragments resulting from the initial UV-induced dissociation are assumed to travel with a constant velocity (calculated from the corresponding TKER values given in the literature^23,26^) before being ionized by the XUV pulse after a certain time delay *τ*. The final TKER is thus a function of the delay, *τ*, and can be calculated as the sum of the TKER of the neutral dissociation, TKER_UV_, and the Coulombic potential energy gained after the two charged fragments, A and B, are created at time *τ*.

$$\text{TKER}(\tau )={\text{TKER}}_{\text{UV}}+\frac{k_{e}q_{A}q_{B}}{r_{\text{AB}}(\tau )}.$$ (1)

Here, *k*_e_, *q*, and *r*_AB_ are the electrostatic constant, fragment charge, and distance between the charges on fragments A and B. The distance *r*_AB_ is calculated for each pump-probe delay. The model assumes an instantaneous charging of both fragmentation partners to the final charge states at the given delay time and a purely Coulombic repulsion between point charges. The TKER_UV_ of the UV-excitation with the higher probability, i.e., I^*^ for CH_3_I and I for DFIB, has been used for the calculations of the two molecules, respectively. As noted in Sec. III A, the assumption that the full TKER_UV_ is added to the Coulomb energy independent of *τ* is an approximation since the fragments do not have this asymptotic energy value for small delays. However, given the difference in magnitude between the Coulomb energy at small delays and the possible change in TKER_UV_, we have neglected the latter for the sake of simplicity. Further details on the calculations are also given in Refs. 56 and 57.

The results are overlaid with the TKER maps in Fig. 3. According to the simulations, the kinetic energy of channel II in all the iodine charge states and for both molecules is best matched when assuming a singly charged co-fragment. This is reasonable since absorption of one XUV photon by the CH_3_ or DFB radical leads, with high probability, to only one charge on this fragment since the photon is absorbed by a valence electron. Core levels of carbon or fluorine are not accessible at a photon energy of 108 eV. In Fig. 3(e), a second weaker feature, labeled II b, is visible in addition to the strong feature, II a. Channel II b has an initially higher but more rapidly decreasing TKER, which could be attributed to Coulomb explosion with a doubly charged co-fragment. However, the agreement with the corresponding simulated TKER curve is not very good. It might be that there is a mixture of DFB^2+^ and DFB^3+^ fragmentation partners, but it seems that, in particular at delays >400 fs, neither of the two simulated curves agree very well with the data. The fact that at least three XUV photoabsorptions are involved in channel II b makes a more quantitative analysis difficult because there is an unknown delay between the ionization events occurring within the XUV pulse duration.

Finally, for the case of DFIB, we can use the Coulomb explosion simulations to investigate the location of the charge on the co-fragment. For that purpose, we take into consideration two limiting cases for the localization of a point charge on the DFB radical, either at the carbon atom located closest to the iodine (grey curves in Fig. 3) or at the carbon atom furthest away (black curves in Fig. 3), also see the inset in Fig. 1(b). In accordance with recent results from a static synchrotron measurement,^58^ the time-resolved TKER data agree much better with the simulations using the largest distance between the charges, i.e., a situation in which two charges are preferentially located at opposite ends of the molecule when the Coulomb repulsion starts. This suggests that the delocalized charge distribution on the phenyl ring is shifted with respect to the center-of-mass of the DFB radical, due to the dipole moment which is induced by the multiply charged iodine ion that is initially in close vicinity of the DFB radical. It is also consistent with an ultrafast charge migration that is instantaneous within the temporal resolution of our experiment, as is expected for purely electronic intramolecular charge rearrangement.^59^ Given the pulse durations of the UV and the XUV pulses in the present experiment, the temporal resolution of the data is not sufficient to draw further conclusions at this point, but experiments with shorter pulse durations may enable studies of such ultrafast charge migration in the near future.

## IV. CONCLUSION AND OUTLOOK

Time-resolved Coulomb explosion imaging of two molecules, iodomethane (CH_3_I) and difluoroiodobenzene (C_6_H_3_F_2_I, DFIB), allowed the influence of the molecular environment on inner-shell ionization to be investigated by an intense, femtosecond XUV pulse at the Free-Electron Laser in Hamburg (FLASH). UV-excitation at 267 nm induced a two-body dissociation, resulting in cleavage of the C–I bond into two neutral fragments. At a photon energy of 108 eV, the XUV probe pulse is absorbed locally at the iodine atom for the case of CH_3_I, while in DFIB, the photoabsorption is less selective. Both molecules exhibit charge transfer from the multiply charged iodine ion to the methyl and phenyl moieties at a short internuclear distance. The timescale of this electron rearrangement is slower in DFIB than in iodomethane because of its slower dissociation velocity.

A non-selective probe pulse ionizing all photofragments can probe the potential energy landscape of a molecule in detail and enables information about both/all partners of the photoexcitation to be obtained, in particular when used in combination with ion-ion coincidences or covariances. In contrast, site-selective absorption at only one fragment, as is the case in CH_3_I, leaves the second partner undetected. However, it facilitates, for example, the creation of a localized source of charge in order to study the electron rearrangement.^18,19,53,60,61^ In the XUV regime, the internuclear charge transfer process can be probed for smaller internuclear distances as compared to X-ray CEI experiments, and therefore, these data provide, in principle, better sensitivity to the orbitals of the intact molecule.

CEI is suitable for investigating dynamics in halogen-substituted benzene following a simple, two-body dissociation, and we plan to extend this technique to other systems and more complex photochemical reactions in the future. The combination with simultaneous femtosecond-resolved electron spectroscopy is a very promising avenue to gain insight into the electronic dynamics that are interconnected with the nuclear motion,^62^ and carrying out complementary pump-probe experiments using either inner-shell or strong-field ionization as a probe can provide valuable additional information on the influence of the probe process.^33,56^ Furthermore, we have presented results indicating that with shorter pump and probe pulse durations and, possibly, in combination with coincident or covariant ion detection, time-resolved CEI might be suitable to directly probe charge localization in polyatomic systems.
